# Realization of a Bilayer Elastic Topological Insulator

**DOI:** 10.1002/advs.202523416

**Published:** 2026-02-03

**Authors:** Chengzhi Ma, Zhiwei Song, Zheyu Cheng, Jiu Hui Wu, Baile Zhang

**Affiliations:** ^1^ School of Mechanical Engineering Xi'an Jiaotong University Xi'an P. R. China; ^2^ School of Physical and Mathematical Science Nanyang Technological University Singapore Singapore; ^3^ State Key Laboratory for Strength and Vibration of Mechanical Structures Xi'an Jiaotong University Xi'an P. R. China; ^4^ State Key Laboratory for Mechanical Behavior of Materials Xi'an Jiaotong University Xi'an P. R. China; ^5^ Centre for Disruptive Photonic Technologies Nanyang Technological University Singapore Singapore

**Keywords:** beam splitting, elastic wave topological insulators, high‐fault‐tolerance elastic waves, interlayer conversions, multi‐topological phases

## Abstract

Elastic waves and their associated devices offer versatile platforms for sensing, metrology, and information processing. The rise of topological materials has enabled unprecedented control of elastic waves in solids, giving rise to elastic wave topological insulators (EWTIs) that host defect‐immune, high‐fault‐tolerance edge states. However, most existing studies remain confined to monolayer configurations, which limit elastic wave propagation and device performance to 2D planes. In contrast, the more promising and practical regime of multilayer elastic wave devices has received little attention. In this work, we experimentally demonstrate a class of bilayer elastic wave topological insulators (BLEWTIs) with high‐fault‐tolerance. By introducing the layer degree of freedom, BLEWTIs exhibit four distinct topological phases, in contrast to the two found in monolayer EWTIs. This enables the construction of multiple types of domain walls with diverse transmission behaviors, such as layer beam splitting. Consequently, we realize high‐fault‐tolerance interlayer converters and beam splitters operating along the normal direction of the 2D plane—features impossible to achieve in monolayer systems. Our findings pave the way for advanced elastic wave applications, including layer‐selective emitters and splitters and multi‐path topological routing, marking a significant step toward the development of compact, high‐performance electromechanical and optomechanical devices.

## Graphical abstract

Bilayer elastic wave topological insulators are experimentally realized, introducing the layer degree of freedom to access four topological phases. This enables diverse domain walls and transmission behaviors, including interlayer conversion and beam splitting. The approach achieves high‐fault‐tolerance converters and splitters along the normal direction, opening avenues for layer‐selective emitters, multi‐path topological routing, and compact, high‐performance elastic wave devices.

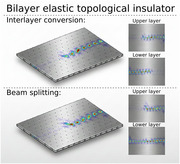

## 1 Introduction

Phonons represent the quantized excitations of elastic waves in solids and acoustic waves in liquids. Owing to their wavelengths comparable to those of electromagnetic waves and frequencies compatible with electronic circuits, photons and phonons can be effectively coupled within integrated circuits [1, 2, 3, 4]. Phononics plays a pivotal role in modern information technologies, with extensive applications in radio‐frequency signal processing [5], navigation [6], sensing [7], non‐destructive testing [8], wave guiding [9, 10], and information processing [11], as well as in advanced military and technological systems [12, 13, 14]. However, elastic wave transport in conventional waveguides inevitably suffers from backscattering in the presence of structural defects and disorder. Recently, significant efforts have been devoted to introducing topology into elastic wave systems [15, 16, 17, 18, 19], which support robust edge modes, inspired by the discovery of topological insulators in condensed matter [20, 21, 22, 23].

Elastic wave topological insulators (EWTIs) have found broad applications; however, their realizations are largely confined to 2D monolayer structures [17, 24, 25, 26, 27, 28, 29, 30, 31, 32, 33, 34, 35], leading to limited elastic wave performance. In contrast, extending EWTIs into three dimensions enables higher‐performance elastic wave devices, paving the way for the miniaturization and on‐chip integration of photon–phonon coupling circuits. In this work, we extend the study of EWTIs into the bilayer regime along the normal direction of the 2D plane. Realizing effective models in elastic wave structures is inherently challenging due to their full‐vector nature and the complicated coupling between longitudinal and transverse components. To overcome this challenge, the upper and lower protruding end faces of the bilayer structure are clamped to eliminate the influence of longitudinal waves, thereby enabling direct control of the transverse waves within the structure. Based on this boundary configuration, we designed and fabricated bilayer elastic structures that supports two distinct types of edge states—layer‐hybridized and layer‐polarized—arising from the additional layer degree of freedom. This bilayer platform serves as a versatile testbed for exploring topological physics in elastic wave systems and opens new avenues for advanced applications such as high‐fault‐tolerance interlayer converters and beam splitters.

The remainder of this article is organized as follows. In Section II, we first provide a detailed description of the primitive cells of bilayer elastic wave topological insulators (BLEWTIs), followed by an analysis of their theoretical models, dispersion relations, and topological phase transitions under triangular prisms with different torsion angles. Next, the dispersion properties of various supercells composed of structures with different topological phases are examined. We then explore elastic wave transmission in BLEWTIs under different conditions, demonstrating high‐performance interlayer conversion and beam splitting. In addition, a missing coupled torsional triangular prism is intentionally introduced into the BLEWTIs to mimic a structural defect, thereby verifying their high‐fault‐tolerance in interlayer conversion and beam splitting. In Section III, we present the experimental verification of these findings. In Section IV, we summarize our work.

## 2 High‐Performance Elastic Wave Transmission in Bilayer Elastic Wave Topological Insulators

### 2.1 Structure Design and Dispersion Relation Analysis

**FIGURE 1:**
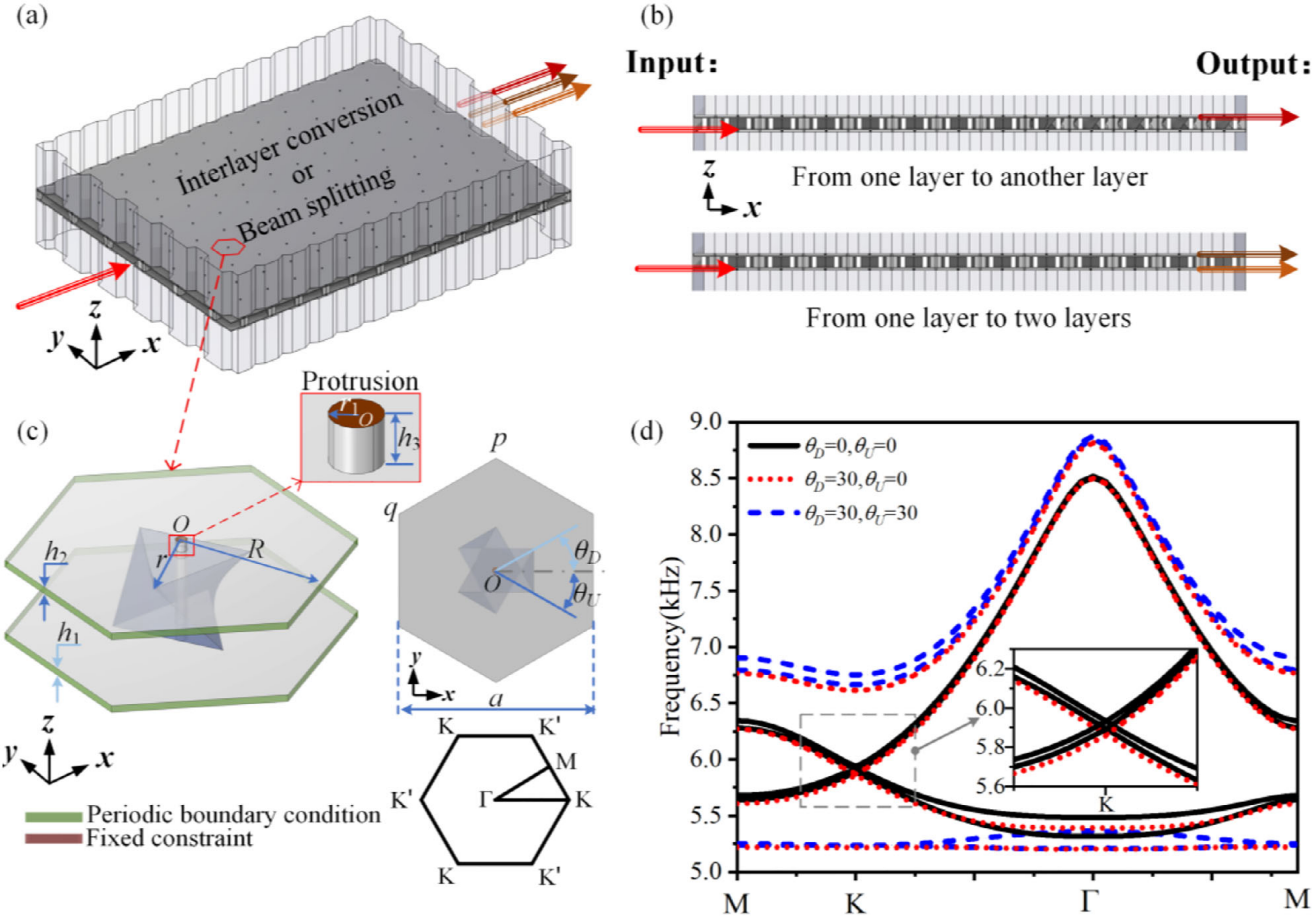
High‐performance elastic wave transmission in bilayer elastic wave topological insulators (BEWTIs). (a) Illustration of interlayer conversion and beam splitting of high‐performance BLEWTIs. (b) Front view of elastic wave transmission. (c) Primitive cell of the BLEWTIs, with the top‐right inset showing its top view and the bottom‐right inset depicting the first Brillouin zone. The brown color indicates the protrusion end face, which is assigned fixed‐constraint boundary conditions. (d) Band structure of the primitive cell under different coupling conditions of twisted triangular prisms.

This study demonstrates that BLEWTIs are achieving lossless, high‐precision elastic wave transmission through bilayer metal plates with high‐fault‐tolerance in Figure 1a. The system supports interlayer conversion as shown in Figure 1b, where the wave from the lower layer is transmitted to the upper layer, and beam splitting as shown in the lower part of Figure 1b, where the single wave is divided into two.

The primitive cell of the BLEWTIs is first constructed, as shown in Figure 1c. It consists of two thin metal plates with twisted triangular prisms sandwiched between them. The angle between the lower end of the triangular prism and the lower metal plate is denoted as θ_*D*_, while the angle between the upper end of the triangular prism and the upper metal plate is denoted as θ_*U*_, as illustrated in the top‐right inset of Figure 1c. The protruding end faces at the top and bottom of the metal plates are fixed (as indicated by the brown color), eliminating the P‐waves and ensuring that all waves propagating in the plates are S‐waves, thereby avoiding the impact of P‐waves on transmission [36]. The boundaries of the upper and lower hexagonal plates along the *x–y* plane are subject to periodic boundary conditions (as indicated by the green color), while all other boundaries except the end faces of protrusions are free. The bottom‐right inset shows the first Brillouin zone (FBZ). The lattice constant of the primitive cell is *a* = 40 mm, and the center point of the unit cell is *O*. The metal plates have thicknesses of *h*_1_ = *h*_2_ = 1 mm, the distance from the center *O* to one side of the hexagonal plate is *R* = 20 mm, and the distance to one edge of the triangular prism is *r* = 9 mm. The height and radius of the top and bottom protrusions are *h*_3_ = 1.5 mm and *r*_1_ = 0.8 mm, respectively. The entire bilayer structure is made of aluminum, with mass density ρ = 2700 kg/m^3^, Young's modulus *E* = 70 GPa, Poisson's ratio υ=0.33, and Damping loss factor η = 0.0002.

To construct the theoretical model of BLEWTIs, we employ the *k* · *p* perturbation approach previously developed for monolayer structures [31]. In monolayer structures with regular triangular protrusions and rotation angle θ = 0°, the system exhibits *C*_3*v*_ symmetry and supports deterministic double degeneracy at K points of the FBZ. Symmetry analysis indicates that the perturbative Hamiltonian produces a linear dispersion relation with slope ± *v_D_* near the Dirac frequency ω_*D*_. Slight rotations of the triangular protrusions open the conical degeneracy at K points, and the perturbative Hamiltonian can then be written as:

(1) $$\delta H^{\prime }=\left[\begin{array}{cc} \omega_{p}^{2}-\omega_{D}^{2} & 2\omega_{D}v_{D}\left(\kappa_{x}-i\kappa_{y}\right)\\ 2\omega_{D}v_{D}\left(\kappa_{x}+i\kappa_{y}\right) & \omega_{q}^{2}-\omega_{D}^{2} \end{array}\right]$$

Here, the basis functions are chosen as the degenerate valley vortex states $\left\{\psi_{p}^{0},\psi_{q}^{0}\right\}$ at θ = 0°. The terms ω_*i*_ (with *i* = {*p*, *q*}) correspond to the frequencies of the non‐degenerate valley vortex states when θ ≠ 0°, and (κ_*x*_,κ_*y*_) denotes the wave vector deviation from the K point. The mass terms $\omega_{p}^{2}-\omega_{D}^{2}\approx 2\eta \omega_{D}\gamma$ and $\omega_{q}^{2}-\omega_{D}^{2}\approx -2\eta \omega_{D}\gamma$ are proportional to the small rotation angle θ, with the coefficient η extracted from the angle‐dependent bandgap [31, 36, 37].

We now consider BLEWTIs. In the absence of interlayer coupling, the basis is taken as the four eigenstates of the BLEWTIs with twisting angles θ_*D*_ = θ_*U*_ = 0°, denoted as $\left\{\psi_{i}^{0}\right\}$, where *i* = {*p_D_*,*q_D_*,*p_U_*,*q_U_*}, and the subscripts *D* and *U* indicate the lower and upper layers, respectively. For small values of θ_*D*_ and θ_*U*_, the Hamiltonian of the BLEWTIs without interlayer coupling is given by the direct sum of the Hamiltonians of the individual layers:

(2)

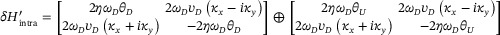




When interlayer coupling is introduced, a new basis is constructed as linear combinations of the original basis states:

(3) $$\left\{\begin{array}{c} \psi_{p,S}^{0}=\frac{\psi_{p_{D}}^{0}+\psi_{p_{U}}^{0}}{\sqrt{2}}\\ \psi_{p,A}^{0}=\frac{\psi_{p_{D}}^{0}-\psi_{p_{U}}^{0}}{\sqrt{2}} \end{array}\right.\;\;\mathrm{and}\;\;\left\{\begin{array}{c} \psi_{q,S}^{0}=\frac{\psi_{q_{D}}^{0}+\psi_{q_{U}}^{0}}{\sqrt{2}}\\ \psi_{q,A}^{0}=\frac{\psi_{q_{D}}^{0}-\psi_{q_{U}}^{0}}{\sqrt{2}} \end{array}\right.$$

Here, the subscripts *S* and *A* indicate “symmetry” and “anti‐symmetry” with respect to the horizontal reflection σ_*h*_. The reflection operator, which satisfies $\sigma_{h}^{2}=1$, $\sigma_{h}\psi_{p_{D}}^{0}=\psi_{p_{U}}^{0}$, and $\sigma_{h}\psi_{q_{D}}^{0}=\psi_{q_{U}}^{0}$, belongs to the point group *D*_3*h*_ of the BLEWTIs with θ_*D*_ = θ_*U*_ = 0°. In this new basis, the Hamiltonian of the BLEWTIs with interlayer coupling is diagonal, with diagonal entries $\left\{\omega_{p,S}^{2},\omega_{p,A}^{2},\omega_{q,S}^{2},\omega_{q,A}^{2}\right\}$. Owing to the equivalence between *p* and *q*, we have ω_*p*,*S*_ = ω_*q*,*S*_ ≡ ω_*S*_ and ω_*p*,*A*_ = ω_*q*,*A*_ ≡ ω_*A*_. Consequently, on the original basis, the perturbation term arising from interlayer coupling is given by:

(4)

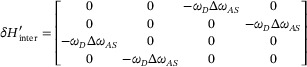




Here, $\Delta \omega_{AS}=\frac{\omega_{A}^{2}-\omega_{S}^{2}}{2\omega_{D}}\approx \omega_{A}-\omega_{S}$ denotes the frequency bandgap between the antisymmetric and symmetric modes. Note that the twisting of the scatterers is not considered for the interlayer coupling, as it contributes only a second‐order correction to the eigenfrequencies. By combining intralayer and interlayer couplings, the total perturbative Hamiltonian of the BLEWTIs (scaled by 2ω_*D*_) is obtained as 
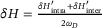
, which satisfies the eigenvalue equation δ*H*ψ = δωψ, where δω represents the frequency deviation relative to ω_*D*_.

Using the Pauli matrices, with σ_*i*_ representing the valley pseudospin and the layer pseudospin *s_i_*, the perturbative Hamiltonian can be compactly expressed as:

(5) $$\delta H=v_{D}\left(\kappa_{x}\sigma_{x}+\kappa_{y}\sigma_{y}\right)+\eta \left(\alpha s_{z}+\beta \right)\sigma_{z}-\Delta_{c}s_{x}$$

Here, $\alpha =\frac{\theta_{D}-\theta_{U}}{2}$, $\beta =\frac{\theta_{D}+\theta_{U}}{2}$, and $\Delta_{c}=\frac{\Delta \omega_{AS}}{2}$. In particular, the term ηα*s_z_* σ_*z*_ simulates the spin‐orbit coupling in the QSHE, while Δ_*c*_ *s_x_* it corresponds to the Zeeman term in electronic systems. For the BLEWTIs studied here, *v_D_* and η depend on the size of the coupled twisted triangular prism, whereas Δ_*c*_ is determined by the geometry of the connectors linking the two thin metal plates.

For brevity, we introduce the substitutions $k_{x}=\frac{v_{D}\kappa_{x}}{\eta }$, $k_{y}=\frac{v_{D}\kappa_{y}}{\eta }$, and $h=\frac{\Delta_{c}}{\eta }$, allowing the Hamiltonian to be rewritten as:

(6) $$\delta H=\eta \left[\left(k_{x}\sigma_{x}+k_{y}\sigma_{y}\right)+\left(\alpha s_{z}+\beta \right)\sigma_{z}-hs_{x}\right]$$

Solving the eigenvalue equation δ*H*ϕ_*n*_ = δω_*n*_ϕ_*n*_ in cylindrical coordinates (*k*, θ), with $k=\sqrt{k_{x}^{2}+k_{y}^{2}}$ and $\theta =\arg(k_{x}+ik_{y})$, we obtain the dispersion relations for the four energy bands: δω_1,2_ = −η*f*_1,2_ and δω_3,4_ = η*f*_2,1_, where the subscripts indicate the band ordering, with $f_{1,2}=\sqrt{k^{2}+h^{2}+\alpha^{2}+\beta^{2}\pm 2\chi }$ and $\chi =\sqrt{\alpha^{2}\beta^{2}+\beta^{2}h^{2}+h^{2}k^{2}}$.

For these BLEWTIs, the elastodynamic boundary‐value problem is solved as follows:

(7) $$\left(\lambda +2\mu \right)\nabla \left(\nabla \cdot u\right)-\mu \nabla \times \left(\nabla \times u\right)=-\rho \omega^{2}u$$

Here **u** is the displacement vector field, and ω is the characteristic frequency. λ and μ are the Lamé constants of the medium, and ρ is the mass density. Equation (7) is solved on the primitive cell with periodic boundary conditions using COMSOL Multiphysics.

By scanning the wave vector *k* along the path M‐K‐Γ‐M of the FBZ, the corresponding eigenvalues ω(*k*), eigen displacement vectors **u**, and dispersion relations are obtained, as shown in Figure 1d. The inset provides an enlarged view of the region within the gray dashed box. Several observations can be made: when θ_*D*_ = 0°, θ_*U*_ = 0°, the band structure exhibits a double Dirac cone at the K point, with no bandgap present; when θ_*D*_ = 30°, θ_*U*_ = 0°, one Dirac cone—dominated by the eigenmodes of the lower layer—opens, forming a bandgap, while the other Dirac cone, dominated by the upper layer, remains intact; and when θ_*D*_ = 30°, θ_*U*_ = 30°, both Dirac cones open, resulting in a bandgap between them. These results indicate that the rotation angles of the triangular prisms play a decisive role in determining the bandgap properties of the primitive cell.

In the simulation, the primitive cell is constructed using the aforementioned structure parameters and defined as a solid mechanics domain. The end faces of the protrusions on both the upper and lower thin plates are assigned fixed constraints (as indicated by the brown color in Figure 1c). Boundaries along the *x–y* plane are subjected to periodic boundary conditions (as indicated by the green color in Figure 1c), while all other boundaries are treated as free. Different regions are respectively meshed by using free tetrahedral grids.

### 2.2 Topological Phase Transitions and Dispersion

**FIGURE 2:**
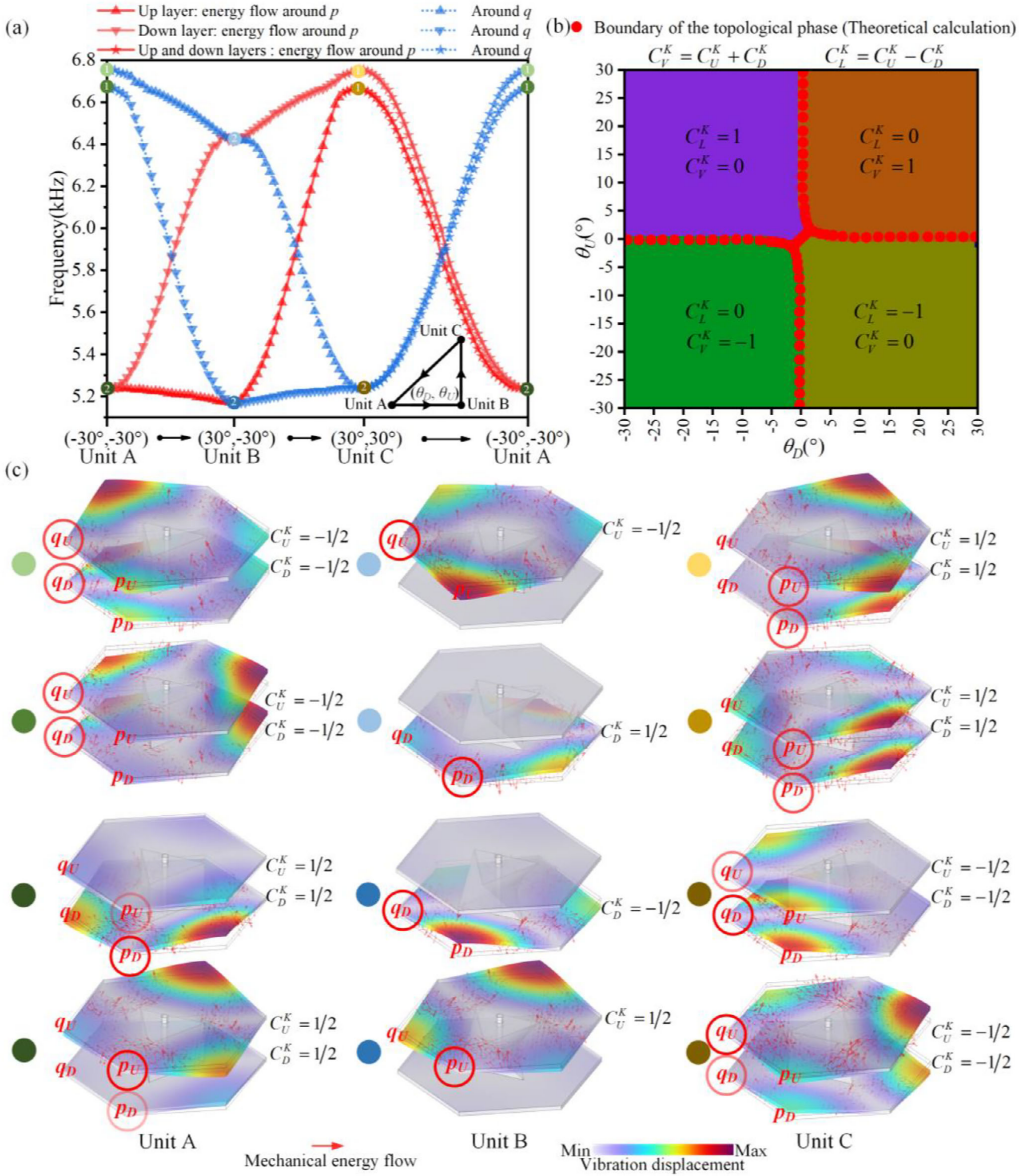
Illustration of topological phase transition as the twisting angles varies. (a) Evolution of the Dirac point at K with variation of the twisting angles θ_*D*_ and θ_*U*_ shown in the bottom‐right inset. The numbers inside the colored circles indicate the corresponding eigenmode count. (b) Distribution of topological phases under different twisting angles. Red dots indicate theoretically calculated boundaries between distinct topological phases. (c) Eigen displacement modes at the positions marked by colored circles in (a). Red arrows represent the magnitude and direction of mechanical energy flow, while red circles highlight energy concentration at positions *p* and *q*, with color intensity representing concentration strength.

We further investigate the topological phase transitions of the primitive cell under different twisting angles of the triangular prisms, denoted as θ_*D*_ and θ_*U*_. As shown in the bottom‐right inset in Figure 2a, the twisting angles θ_*D*_ and θ_*U*_ vary along the indicated path. The evolution of the double Dirac points at the K point is illustrated in Figure 2a. Upright triangles represent eigenmodes primarily localized in the upper layer, inverted triangles correspond to modes mainly in the lower layer, and pentagrams denote modes distributed across both layers. Solid red lines indicate that the mechanical energy flow of the eigenmodes is predominantly concentrated at position *p*, while dashed blue lines indicate concentration at position *q*.

It can be observed that when θ_*D*_ = −30°, θ_*U*_ = −30° (Unit A), the Dirac points are dominated by the open upper and lower layers. The eigenmodes corresponding to the green dots are extracted, with the numbers inside the dots indicating the number of associated modes. As shown in Figure 2c, the two upper bands correspond to mechanical energy flows concentrated at position *q*, distributed across both the upper and lower layers. Position *q* in the upper layer is denoted as *q_U_*, and in the lower layer as *q_D_*. Red circles mark the locations of mechanical energy flow, with color intensity representing the strength of distribution. The two eigenmodes in the lower band have nearly overlapping frequencies; one mode exhibits mechanical energy flow predominantly at *p_D_*, while the other is concentrated mainly at *p_U_*.

Next, with θ_*U*_ = −30° held constant, as varies θ_*D*_ from − 30° to 30° (Unit B), the band corresponding to the eigenmodes of the lower thin plate first closes and then reopens. The eigenmode diagrams corresponding to the blue dots show that the mechanical energy distribution in the upper layer remains unchanged, while the positions *p* and *q* in the lower layer are swapped. Subsequently, with θ_*D*_ = 30° held constant, as θ_*U*_ varies from − 30° to 30° (Unit C), the band corresponding to the upper thin plate first closes and then reopens. The eigenmode diagrams corresponding to the ochre points indicate that the mechanical energy distribution in the lower layer remains unchanged, while positions *p* and *q* in the upper layer are swapped. Finally, when both θ_*D*_ and θ_*U*_ return from 30° back to − 30°, returning to Unit A, the mechanical energy flow positions *p* and *q* in both the upper and lower layers are exchanged.

The topological phases of the upper and lower thin metal plates in the primitive cell are calculated separately. Unlike the global characteristics of the QHE and QSHE, which are defined across the entire Brillouin zone, the valley Hall effect (VHE) relies on topological properties that are local, being effective only near the valleys. In other words, the topological characteristic in the VHE is confined to narrow regions surrounding the valley points.

The topological properties of the lattice in the VHE can be characterized by the valley Chern number, *C_v_*, which is a variant of the well‐known Chern number *C_n_* used in the QHE. In this primitive cell, composed of upper and lower thin metal plates, the primary eigenmodes in each plate occupy specific energy bands. The valley Chern number *C_v_* for the *n*‐th energy band is then given by [38]:

(8) $$2\pi C_{\nu }=\int \Omega_{n}\left(k\right)d^{2}k$$

The Berry curvature of the *n‐*th mode is given by:

(9) $$\Omega_{n}\left(k\right)=\nabla_{k}\times \langle u_{n}\left(k\right)|i\nabla_{k}|u_{n}\left(k\right)\rangle \cdot \hat{z}$$

Here, *k* is the wave vector, *u_n_* (*k*) is the displacement of the *n*‐th band as a function of *k*, $\hat{z}$ is the unit vector perpendicular to the plane, and ∇_*k*_ denotes the gradient operator. Using a finite‐difference formula in space *k_x_* − *k_y_*, ∇_*k*_|*u_n_* (*k*)〉 it can be computed. If we treat the eigenmode as a vector with 3*N* scalar elements (where *N* is the number of nodes in the finite element model), then ∇_*k*_|*u_n_* (*k*)〉 it is a second‐order tensor, with the first index corresponding to the number of nodes and the second index representing derivatives along *k_x_* or *k_y_*. The inner product integral 〈*u_n_* (*k*)|*i*∇_*k*_|*u_n_* (*k*)〉 (the volume integral over the entire cell) eliminates the virtual index (node number), yielding a 2D vector field in space *k_x_* − *k_y_*, known as the Berry connection. Finally, taking the curl in space as in Equation (9) gives the Berry curvature.

The area integral in Equation (8) is evaluated in *k*‐space over a small region surrounding a valley (K or K'). This differs from the Chern number *C_n_* commonly used in the QHE, which requires integrating the Berry curvature over the entire Brillouin zone. Notably, due to the distribution of Berry curvature in *k*‐space for the current lattice, *C_n_* it vanishes when integrated over the entire Brillouin zone, which is why VHE‐based systems are considered topologically trivial in the QHE sense. However, the valley Chern number *C_v_* computed over a small neighborhood around the valley attains a finite value, endowing the system with a locally nontrivial topology in *k*‐space.

The right‐hand side of Equation (8) represents a quantized value known as the topological charge. This quantity is directly related to the bulk‐edge correspondence [39], which predicts the number of edge states arising from specific topological properties of the bulk bands. Previous studies have shown that, for small twisting angles, the Berry curvature is strongly localized near the valley, and the valley Chern number rapidly converges to $C_{\nu }=\pm \frac{1}{2}$ within a small region around K (or K'). Furthermore, opposite perturbations yield opposite signs of *C*_ν_, indicating distinct topological phases.

Here, we introduce the layer‐polarized valley Chern number, $C_{D/U}^{K}$, to characterize the two bands below the omnidirectional bandgap, where *U* and *D* correspond to recombined states confined to the upper and lower layers, respectively. This approach has been previously developed in electronic systems [40, 41, 42].

For the current unit cell, composed of upper and lower thin metal plates connected by twisted prisms, the Berry curvature is calculated within a finite square region around the K point using Equation (8), based on the intrinsic vibration modes of each plate. Integrating the Berry curvature yields the valley Chern numbers for Units A, B, and C, as shown in Figure 2c. The topological phases for the upper and lower layers are denoted as $C_{U}^{K}$ and $C_{D}^{K}$, respectively. It is observed that when one layer has $C_{D/U}^{K}=\frac{1}{2}$ a valley Chern number, applying the opposite twisting angle to that layer produces a valley Chern number of $C_{D/U}^{K}=-\frac{1}{2}$, thereby generating a distinct topological phase. This confirms that the two configurations of the same thin metal plate are topologically distinct. The mechanical energy flow concentrated at position *p* in the upper layer corresponds to $C_{U}^{K}=\frac{1}{2}$, while at position *q* it corresponds to $C_{U}^{K}=-\frac{1}{2}$. In the lower layer, concentration at position *p* corresponds to $C_{D}^{K}=\frac{1}{2}$, and at position *q* it corresponds to $C_{D}^{K}=-\frac{1}{2}$. Furthermore, as shown in the topological phase diagram in Figure 2b, two types of quantized topological invariants are defined to characterize the valley‐projected topological phases: $C_{V}^{K}=C_{U}^{K}+C_{D}^{K}$ and $C_{L}^{K}=C_{U}^{K}-C_{D}^{K}$. The former is a natural bilayer extension of the valley Chern number commonly used in monolayer systems [37], while the latter encodes the layer‐specific information.

Further theoretical calculations based on the above model accurately predict the boundaries of the topological phases. As discussed previously, topological phase transitions are associated with the closure of the omnidirectional bandgap between the two inner bands (the 2nd and 3rd bands). This occurs when the condition δω_2_ = δω_3_ is satisfied, i.e., *f*_2_ = 0, from which the expression for the wave vector *k* can be derived:

(10) $$k=\sqrt{h^{2}-\alpha^{2}-\beta^{2}\pm 2\sqrt{\alpha^{2}\left(\beta^{2}-h^{2}\right)}}$$

Real solutions for *k* are obtained when either α = 0 or β^2^ ≥ *h* ^2^ is satisfied.

When α = 0the solution $k=\sqrt{h^{2}-\beta^{2}}$ is obtained within the range − *h* ≤ β ≤ *h*. When β^2^ ≥ *h* ^2^a zero‐wavevector solution *k* = 0 can be obtained via $k=\sqrt{-{\left(\sqrt{\beta^{2}-h^{2}}\pm \left|\alpha \right|\right)}^{2}}$, provided that $\beta =\pm \sqrt{h^{2}+\alpha^{2}}$ is satisfied.

By substituting $\alpha =\frac{\theta_{D}-\theta_{U}}{2}$ and $\beta =\frac{\theta_{D}+\theta_{U}}{2}$ into the relevant equation above, when θ_*D*_ = θ_*U*_ within the range − 2*h* ≤ θ_*D*_ + θ_*U*_ ≤ 2*h*, the solution $k=\sqrt{h^{2}-{\left(\frac{\theta_{D}+\theta_{U}}{2}\right)}^{2}}$ is obtained. This corresponds to the straight phase boundary marked by red dots at the center of Figure 2b.

When (θ_*D*_ + θ_*U*_)^2^ ≥ 4*h* ^2^a zero‐wavevector solution *k* = 0 can be obtained via $k=\sqrt{-{\left[\sqrt{{\left(\frac{\theta_{D}+\theta_{U}}{2}\right)}^{2}-h^{2}}\pm \left|\frac{\theta_{D}-\theta_{U}}{2}\right|\right]}^{2}}$, provided that $\frac{\theta_{D}+\theta_{U}}{2}=\pm \sqrt{h^{2}+{\left(\frac{\theta_{D}-\theta_{U}}{2}\right)}^{2}}$ is satisfied. This corresponds to the curved phase boundaries marked by red dots in Figure 2b.

The different colored regions in Figure 2b represent the various topological phases obtained from simulations. The interfaces between these regions show excellent agreement with the topological phase boundaries predicted by theoretical calculations, as indicated by the red dots.

**FIGURE 3:**
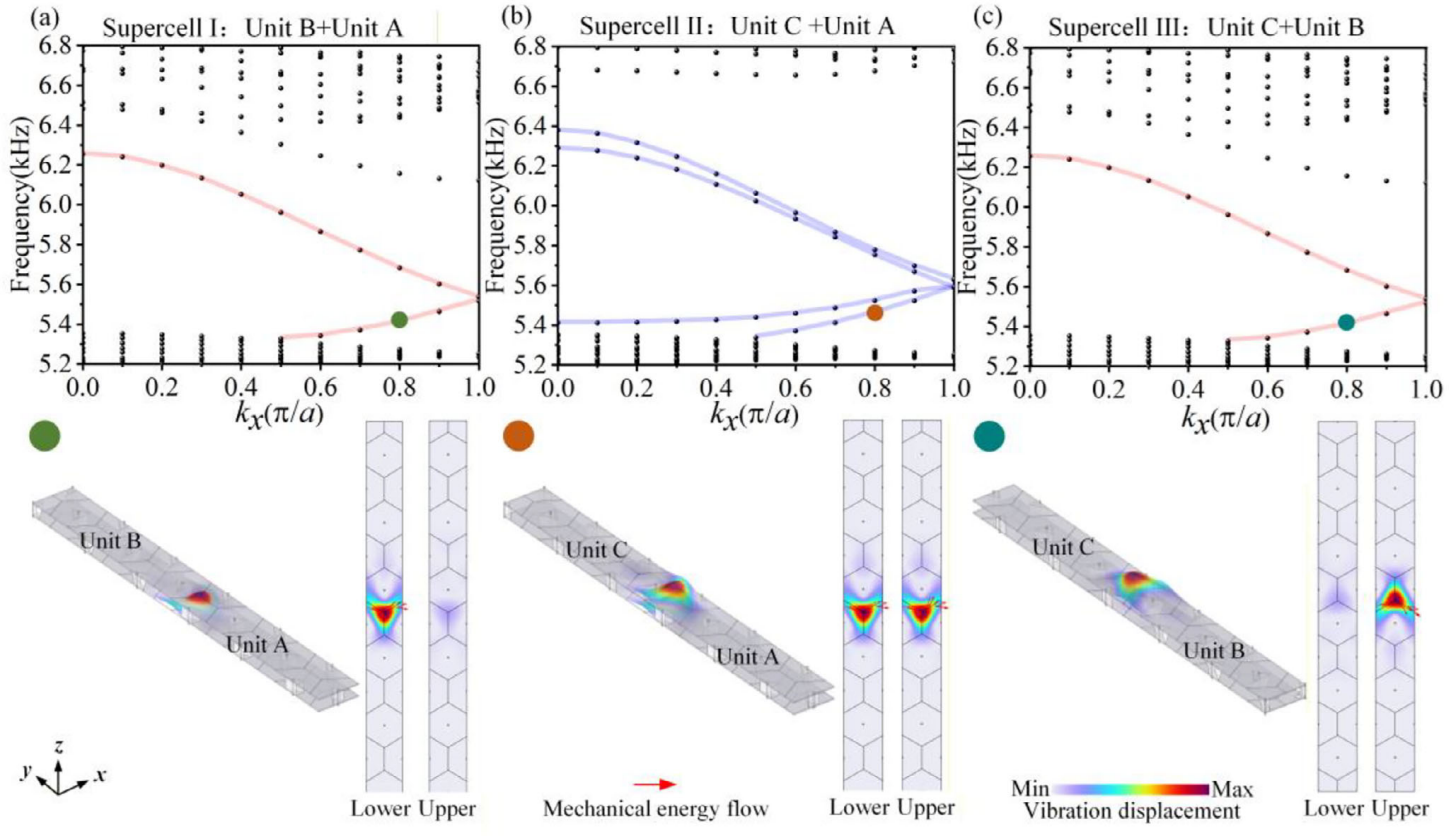
Dispersion of the supercells. (a) Band structure of the supercell composed of Unit B and Unit A. The lower inset shows the eigenmode displacement field at the green dot, with red arrows indicating the mechanical energy flow. (b) Band structure of the supercell composed of Unit C and Unit A. The lower inset shows the eigenmode displacement field at the orange dot. (c) Band structure of the supercell composed of Unit C and Unit B. The lower inset shows the eigenmode displacement field at the cyan dot.

Topological interface states in the BLEWTIs are constructed using different combinations of Unit A, Unit B, and Unit C, as illustrated in Figure 3. Supercell I, composed of Unit B and Unit A as shown in Figure 3a, exhibits topological interface bands within the bandgap. The interface state corresponding to the green dot at *k_x_* = 0.8π/*a* shows vibration highly localized at the interface in the lower metal plate. The inset displays the intrinsic vibration modes in both metal plates, with red arrows indicating the mechanical energy flow. Similarly, Supercell II, composed of Unit C and Unit A as shown in Figure 3b, also shows topological interface bands within the bandgap, with the interface state at the orange dot localized at the interface in both upper and lower metal plates. Supercell III, composed of Unit C and Unit B as shown in Figure 3c, exhibits interface bands within the bandgap, with vibration localized at the interface in the upper metal plate, corresponding to the cyan dot. In the simulation, periodic boundary conditions are applied along the *x*‐direction of the supercell, while the *y*‐direction boundary is set as a free boundary condition. All other settings are the same as those for the primitive cell.

Based on the valley Chern numbers of the constituent primitive cells, the absolute value of the difference across the interface where vibration is concentrated is $|\Delta C_{\nu }^{K}|=1$. According to the bulk‐boundary correspondence [39], this confirms the presence of edge modes at the interfaces, corresponding to the observed topological interface states.

### 2.3 Interlayer Conversion and Beam Splitting of High‐performance Elastic Waves in Bilayer Elastic Wave Topological Insulators

#### 2.3.1 Interlayer Conversion of High‐performance Elastic Waves

**FIGURE 4:**
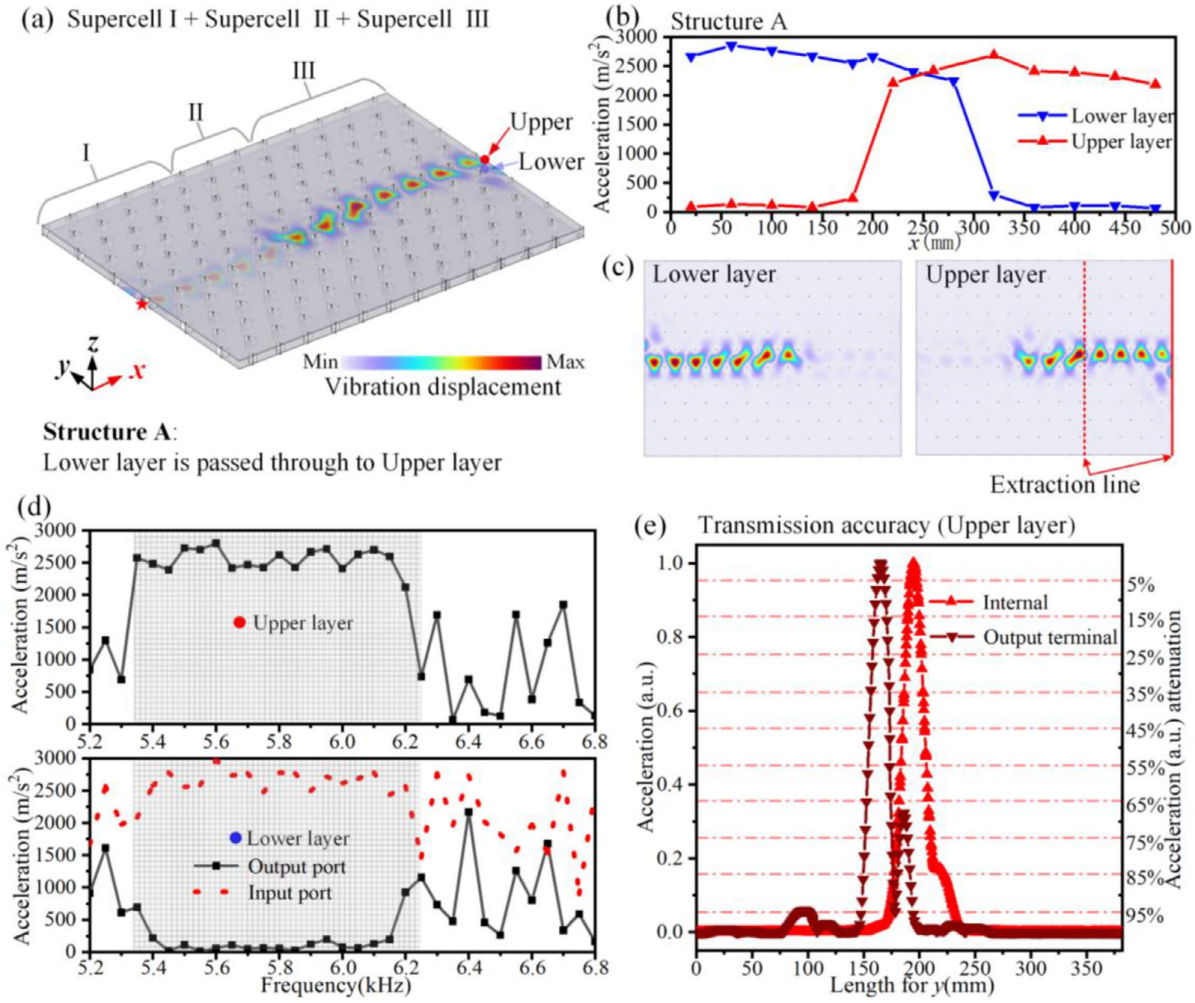
Interlayer conversion of high‐performance elastic waves. (a) Structure A: Interlayer conversion of high‐performance elastic waves. (b) The vibration acceleration along the *x*‐axis in the lower and upper metal plates of the structure in (a). (c) The vibration displacement distribution in the upper and lower layers. (d) The vibration acceleration at the output ports of the upper and lower metal plates under different frequencies. The grid‐shaded region represents the frequency range of the topological interface modes. (e) Transmission accuracy in Structure A. The “up triangles” and “down triangles” denote the normalized acceleration extracted along the dotted line and the dashed line in panel (c), respectively.

Based on the topological interface states of the different supercells, finite‐sized Supercells I, II, and III are combined to construct Structure A of the BLEWTIs, as shown in Figure 4a. A point vibration excitation with a frequency range of 5.2–6.8 kHz is applied to the lower layer (red pentagram). All parts of the structure are assigned free boundary conditions, except for the protruding end face, which is set as a fixed constraint. The vibration acceleration in the upper layer output port (marked as red dot in Figure 4a), lower layer output port (marked as blue dot in Figure 4a), and input port (marked as star in Figure 4a) are presented in Figure 4d. Within the frequency range of the topological interface modes (the grid‐shaded region), nearly all vibration energy from input port is transmitted to the upper output port, while the lower output port receives negligible energy. The vibration modes at 5.5 kHz are shown in Figure 4c, revealing that the vibration is initially concentrated at the front section of the lower layer interface and subsequently at the rear section of the upper layer interface. The vibration acceleration along the *x*‐axis at the interface positions of the upper and lower plates, as shown in Figure 4b, further confirms high‐performance elastic wave transmission with efficient interlayer conversion from the lower to the upper layer.

The vibration displacement distribution further shows that the topological interface state effectively confines the energy at the interface, with rapid decay into the bulk, indicating that the bulk of the structure behaves as an “insulator”. Transmission accuracy within the structure, as well as at the internal and output terminals, is analyzed and indicated in Figure 4e by “up triangles” and “down triangles,” respectively. To more clearly illustrate the location of the maximum vibration acceleration and its attenuation along the normal direction of transmission, the normalized vibration acceleration is extracted along the red dashed line and the red solid line in Figure 4c, representing positions inside the structure and at the output port, respectively. Both profiles exhibit extremely high transmission accuracy, with 90% of the vibration energy decaying within 0.7*a* (see Table 1 for details). The position of the maximum acceleration at the output terminal exhibits a slight shift of approximately 0.5*a* due to boundary effects—specifically, the distortion of the mode profile at the output terminal relative to that within the structure—yet the system still maintains excellent transmission performance.

**TABLE 1:** Effective range (Λ) corresponding to 90% energy attenuation at different positions in intact and defective Structure A.

Upper Layer	Output Terminal	Internal Position 1	Internal Position 2
Intact Structure A	Λ = 0.572*a*	/	Λ = 0.691*a*
Defective structure A	Λ = 0.675*a*	Λ = 1.798*a*	Λ = 0.784*a*

The fundamental physical origin of the shift in the position of maximum acceleration in transmission accuracy at the boundary is the breaking of translational symmetry. At the output terminal, the wave transitions from a guided mode to a free mode in the surrounding environment. Consequently, the topological interface mode profile becomes distorted at the boundary and differs from its counterpart within the structure. Figure 3 illustrates a 1D periodic supercell structure. Its eigenmode distribution shows that, in the absence of 1D boundaries along the *x*‐direction, the maximum vibrational displacement is located at the center of the supercell. This behavior is consistent with the vibration mode distribution observed within the structure, i.e., far from the boundary, as illustrated in Figure 4c.

**FIGURE 5:**
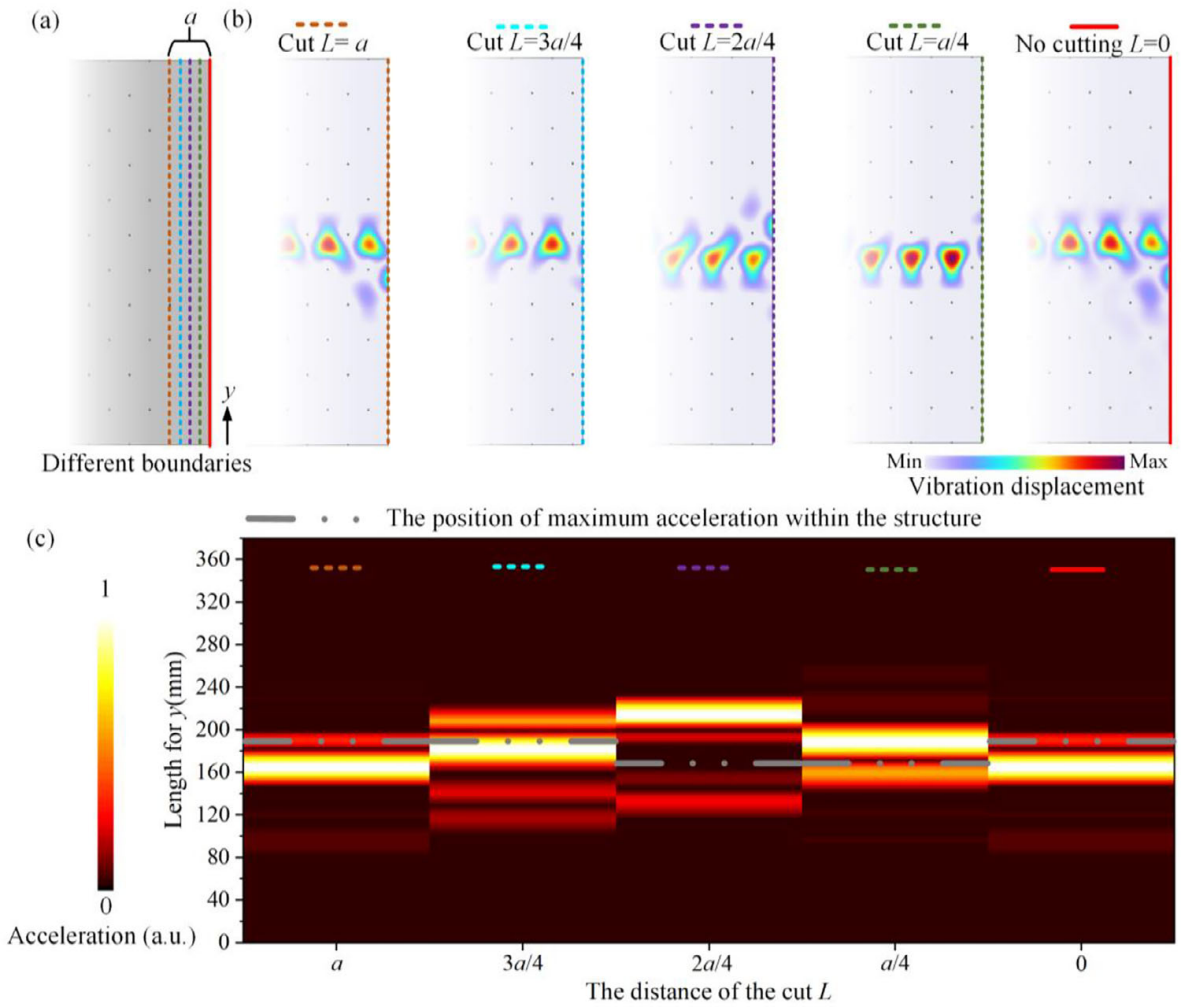
Relationship between the position of the maximum vibration displacement (or acceleration) and the output‐terminal positions. (a) Illustration of the output‐terminal positions. (b) Profiles of vibration displacement for different output terminals at 5.5 kHz. (c) Transmission accuracy for different output terminals. The colored dashed lines correspond to the output‐terminal positions indicated in panel (a). The grey double‐dotted line represents the position of maximum acceleration within the structure, i.e., far from the boundary.

Here, we further investigate the influence of different boundaries on transmission accuracy. Since the supercell structure is periodic with lattice constant *a*, we introduce different output‐terminal positions for Structure A, as illustrated in Figure 5a. Starting from the original boundary, output‐terminal cuts at distances *L*  =  *a*/4, *L*  =  2*a*/4, *L*  =  3*a*/4, and *L*  =  *a* are indicated by green, purple, light‐blue, and orange dashed lines, respectively. For all cases, the structure is excited over a frequency range of 5.2–6.8 kHz by the actuator, and the vibrational displacement distributions in the upper output‐terminal region are extracted; the mode profile at 5.5 kHz is shown in Figure 5b as an example. The results indicate that the vibrational displacement distribution within the structure closely matches the eigenmode distribution of the periodic supercell (Figure 3), confirming that the observed modifications in the mode profiles originate primarily from the boundary.

The transmission accuracy at the output terminal for the different boundary configurations is presented in Figure 5c. To clearly illustrate the peak positions and attenuation behavior, the data are normalized to the respective peak values for each case. Compared with the peak position inside the structure (indicated by the gray double‐dotted line), the peak position at the output terminal exhibits a certain degree of variation; however, its overall range remains confined within 1.5*a*. In addition, the peak position inside the structure also varies along *x*. This is reasonable, as the center of the Bloch function is not required to remain constant. Owing to the protection afforded by periodicity, this shift is relatively small and confined within a range of *a*. Notably, the mode localization at the terminal is reduced for the cases *L*  =  *a*/4 and *L*  =  3*a*/4. In other words, the transmission accuracy decreases in these two cases. This decreases primarily arises because, at these terminations, the boundary lacks a fixed constraint provided by protruding end faces, rendering it closer to a free boundary compared with the other configurations. As a result, the transmission accuracy—defined as the localization degree of vibrational energy along the transmission path—is reduced, indicating that fixed constraints at the protruding end faces enhance transmission accuracy.

#### 2.3.2 Beam Splitting of High‐performance Elastic Wave

**FIGURE 6:**
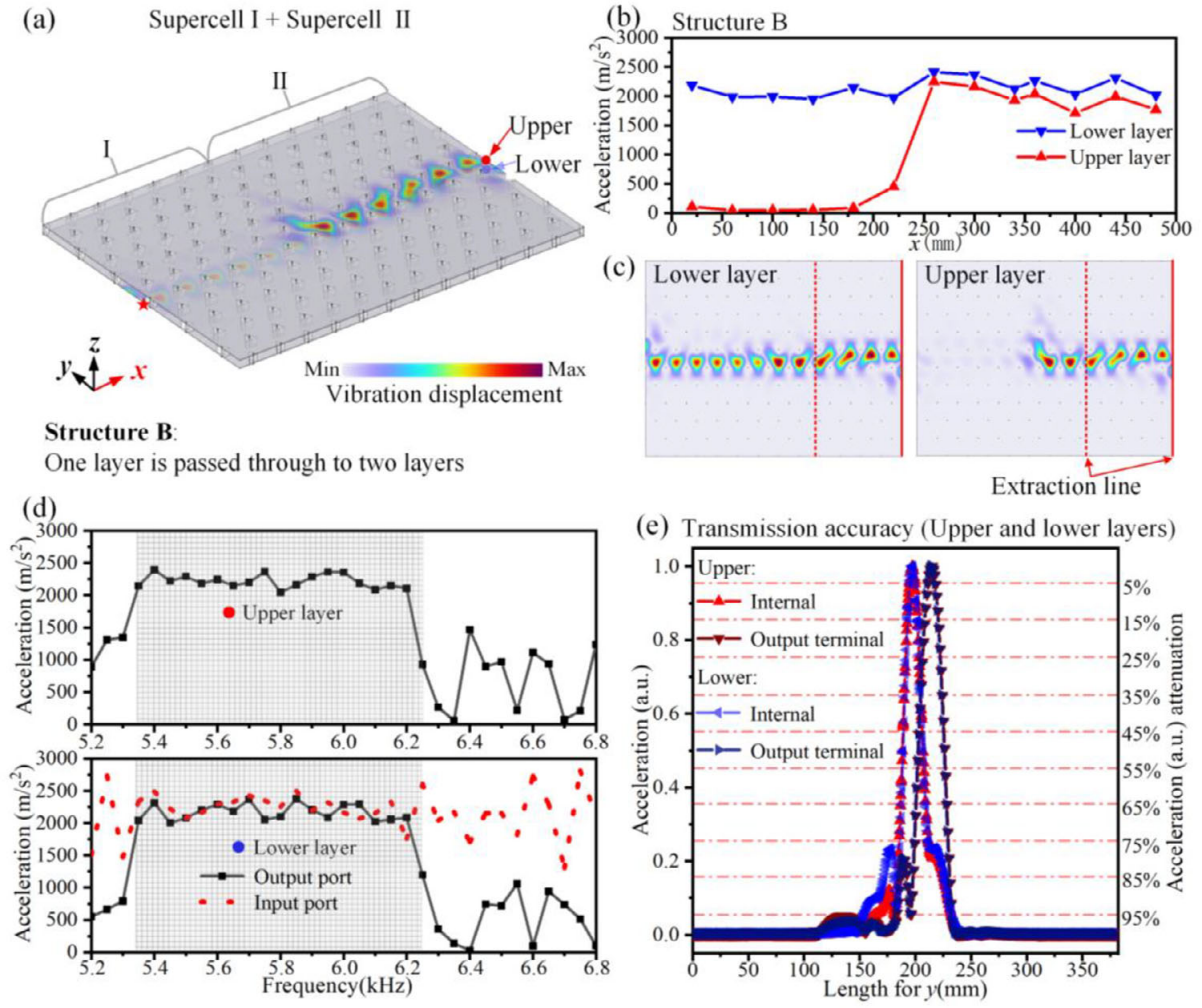
Beam splitting of high‐performance elastic waves. (a) Structure B: Beam splitting of high‐performance elastic waves. (b) The vibration acceleration along the *x*‐axis in the lower and upper metal plates of the structure in panel (a). (c) The vibration displacement distribution in the upper and lower layers. (d) The vibration acceleration at the output ports of the upper and lower metal plates under different frequencies. The grid‐shaded region represents the frequency range of the topological interface modes. (e) Transmission accuracy in Structure B. The “Internal” and “Output terminal” denote the normalized acceleration extracted along the dotted line and the dashed line in panel (c), respectively.

Achieving high‐performance multiplexing of vibration energy is crucial, and realizing beam splitting of elastic waves within multilayer metallic plates is of great significance. Leveraging the topological interface states of different supercells, Structure B of the BLEWTIs is constructed by combining finite‐sized Supercell I and Supercell II, as shown in Figure 6a. The vibration acceleration at the upper and lower output ports is presented in Figure 6d, within the topological interface band of the supercells; both output ports receive vibration energy with negligible attenuation. The vibration modes at 5.5 kHz are shown in Figure 6c, revealing that the energy is highly concentrated along the entire lower interface and in the rear section of the upper interface. The vibration acceleration along the *x*‐axis at the interface of both plates in Figure 6b further confirms high‐performance beam splitting, where the input from the lower layer is simultaneously transmitted to the upper and lower layers, achieving low‐loss energy transmission and efficient splitting of a single beam into two.

The transmission accuracy along the dotted and solid lines is extracted from Figure 6c, with the results shown in Figure 6e. High transmission accuracy is observed in both the upper and lower layers within the structure and at the output terminals, confirming excellent beam‐splitting performance.

### 2.4 High‐Fault‐Tolerance Interlayer Conversion and Beam Splitting of High‐Performance Elastic Waves in Defective Bilayer Elastic Wave Topological Insulators

#### 2.4.1 High‐Fault‐Tolerance Interlayer Conversion of High‐performance Elastic Wave

**FIGURE 7:**
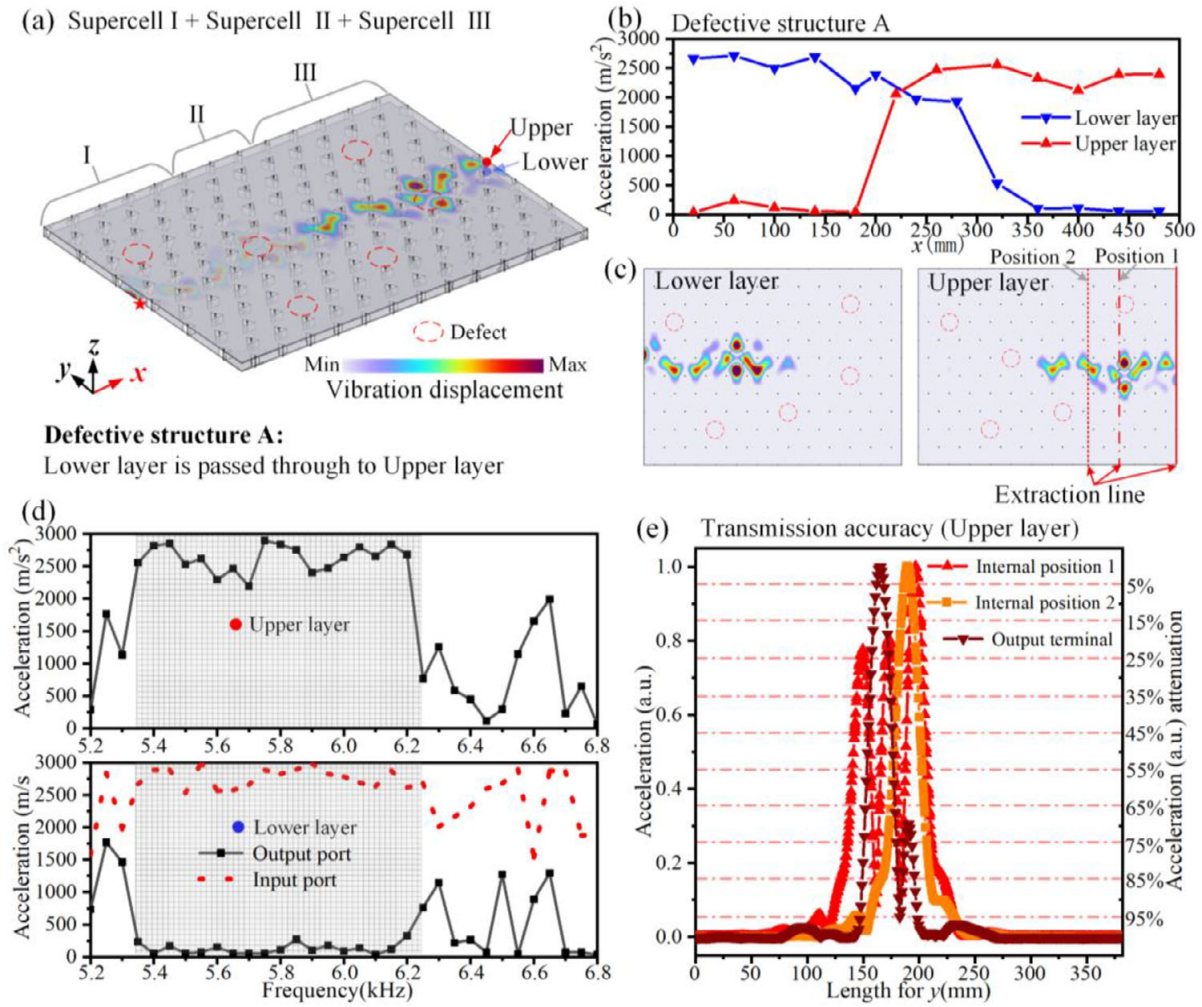
High‐fault‐tolerance interlayer conversion of high‐performance elastic waves. (a) Defective Structure A: High‐fault‐tolerance interlayer conversion of high‐performance elastic waves, where the red dashed circle marks the missing part of the coupled twisted triangular prism. (b) The vibration acceleration along the *x*‐axis in the lower and upper thin metal plates of the structure in (a). (c) The vibration displacement distribution in the upper and lower layers. (d) The vibration acceleration at the output ports of the upper and lower thin metal plates under different frequencies. The grid‐shaded region represents the frequency range of the topological interface modes. (e) Transmission accuracy in defective Structure A. The data are extracted along the “Extraction line” indicated in panel (c).

In practical applications, manufacturing inaccuracies as well as device aging or damage over time are unavoidable, and conventional devices are often highly susceptible to such imperfections. Owing to the inherent stability of topological phases against local perturbations [43], we further examine the high‐fault‐tolerance properties of BLEWTIs by randomly removing several coupled twisted triangular prisms within Structure A. The defect locations are marked by the red dashed circles in Figure 7a, while all other conditions were kept identical to those of Structure A shown in Figure 4a.

We present in Figure 7 physical quantities similar to those shown in Figure 4, including the vibrational acceleration as a function of propagation distance and frequency, as well as the transmission accuracy. The normalized vibrational accelerations are extracted along the red dash‐dotted line, red dashed line, and red solid line in Figure 7c, corresponding to the defect locations, intact regions, and output terminal, respectively. As expected, the vibrational modes are locally perturbed in the vicinity of the missing coupled twisted triangular prisms; however, the overall transmission remains stable. To quantitatively evaluate the influence of defects on transmission accuracy, we define an effective range, Λ, as the distance over which 90% of the vibrational energy is attenuated. Since the vibrational energy is proportional to the square of the acceleration amplitude, this corresponds to a 68.4% reduction in the vibration acceleration amplitude.

We calculate the effective range Λ for the extraction lines shown in Figures 4c and 7c, and the corresponding values are summarized in Table 1. Since internal position 1 corresponds to the defect location, no value is reported for the intact Structure A. After introducing a defect at internal position 1, the effective range Λ at the intact region (internal position 2) and at the output terminal shows only minor variation, whereas a pronounced change is observed at the defect location (internal position 1). This clearly demonstrates that the influence of defects remains highly localized, consistent with the robustness expected from topological interface states, and thus does not degrade the overall transmission accuracy.

#### 2.4.2 High‐Fault‐Tolerance Beam Splitting of High‐Performance Elastic Waves

**FIGURE 8:**
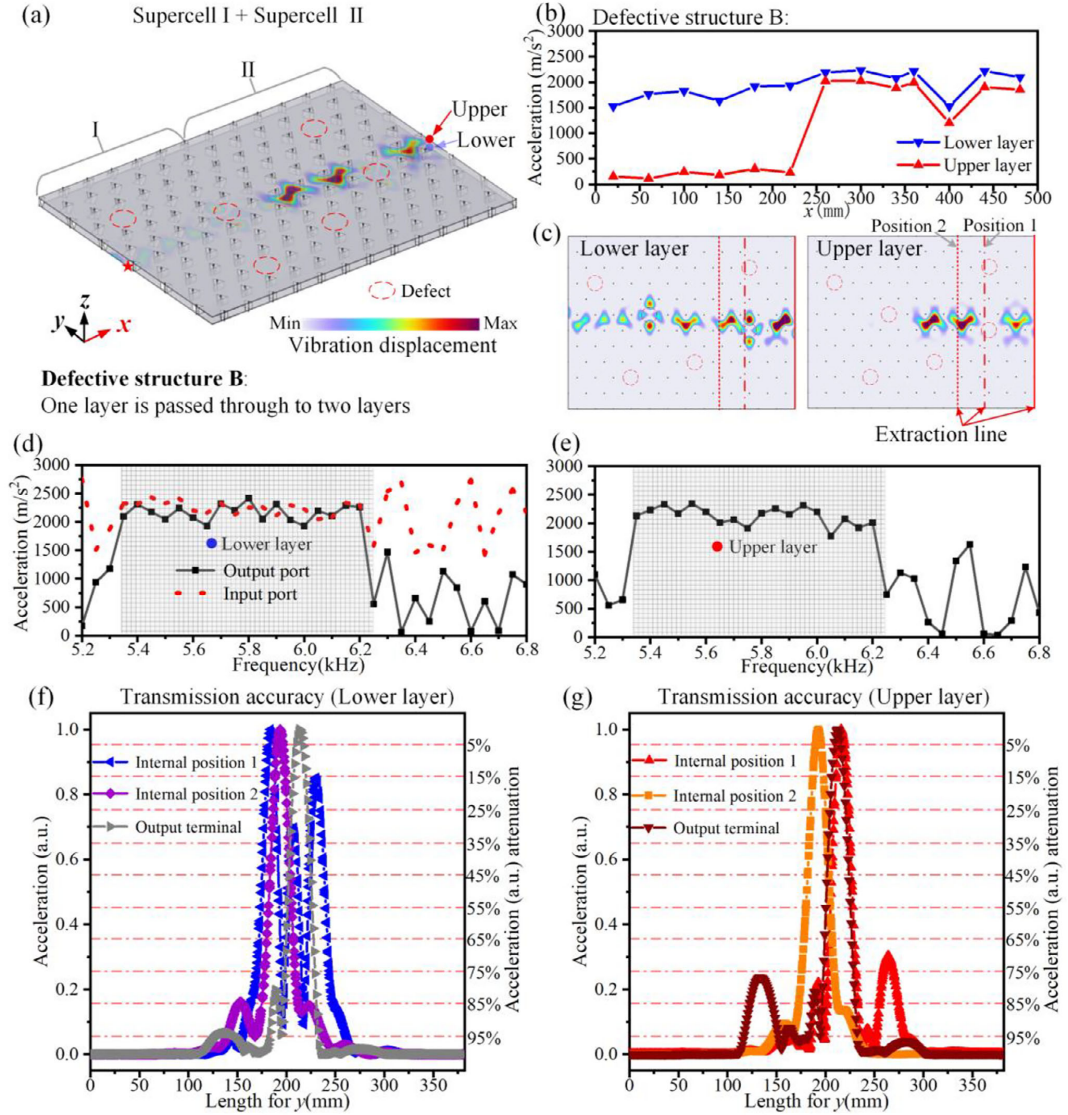
High‐fault‐tolerance beam splitting of high‐performance elastic waves. (a) Defective Structure B: High‐fault‐tolerance beam splitting of high‐performance elastic waves, where the red dashed circle marks the missing part of the coupled twisted triangular prism. (b) The vibration acceleration along the *x*‐axis in the lower and upper thin metal plates of the structure in (a). (c) The vibration displacement distribution in the upper and lower layers. (d) The vibration acceleration at the input and output ports of the lower thin metal plates under different frequencies. (e) The vibration acceleration at the output port of the upper thin metal plates under different frequencies. The grid‐shaded region represents the frequency range of the topological interface modes. (f,g) Transmission accuracy in the lower and upper layers of the defective Structure B. The data are extracted along the “Extraction line” indicated in panel (c).

A similar defective structure B is constructed, as illustrated in Figure 8a. We present in Figure 8 physical quantities similar to those shown in Figure 6, including the vibrational acceleration as a function of propagation distance and frequency, as well as the transmission accuracy. We calculate the effective range Λ for the extraction lines shown in Figures 6c and 8c, and the corresponding values are summarized in Table 2. Data in Table 2 demonstrates that the influence of defects remains highly localized, consistent with the robustness expected from topological interface states, and thus does not degrade the overall transmission accuracy.

**TABLE 2:** Effective range (Λ) corresponding to 90% energy attenuation at different positions in intact and defective Structure B.

Upper Layer	Output Terminal	Internal Position 1	Internal Position 2
Intact Structure B	Λ = 0.711*a*	*/*	Λ = 0.598*a*
Defective structure B	Λ = 0.726*a*	Λ = 0.672*a*	Λ = 0.763*a*
Lower layer	Output terminal	Internal position 1	Internal position 2
Intact Structure B	Λ = 0.711*a*	*/*	Λ = 0.570*a*
Defective structure B	Λ = 0.722*a*	Λ = 1.783 *a*	Λ = 0.723*a*

In conclusion, under the protection of topological states in the bilayer metal plate, both interlayer conversion and beam splitting of high‐performance elastic waves are realized without noticeable degradation in the energy transmission, frequency band, or accuracy, even in the presence of defects. These results demonstrate that the bilayer elastic wave device possesses exceptionally high fault tolerance.

## 3 Experimental Verification

**FIGURE 9:**
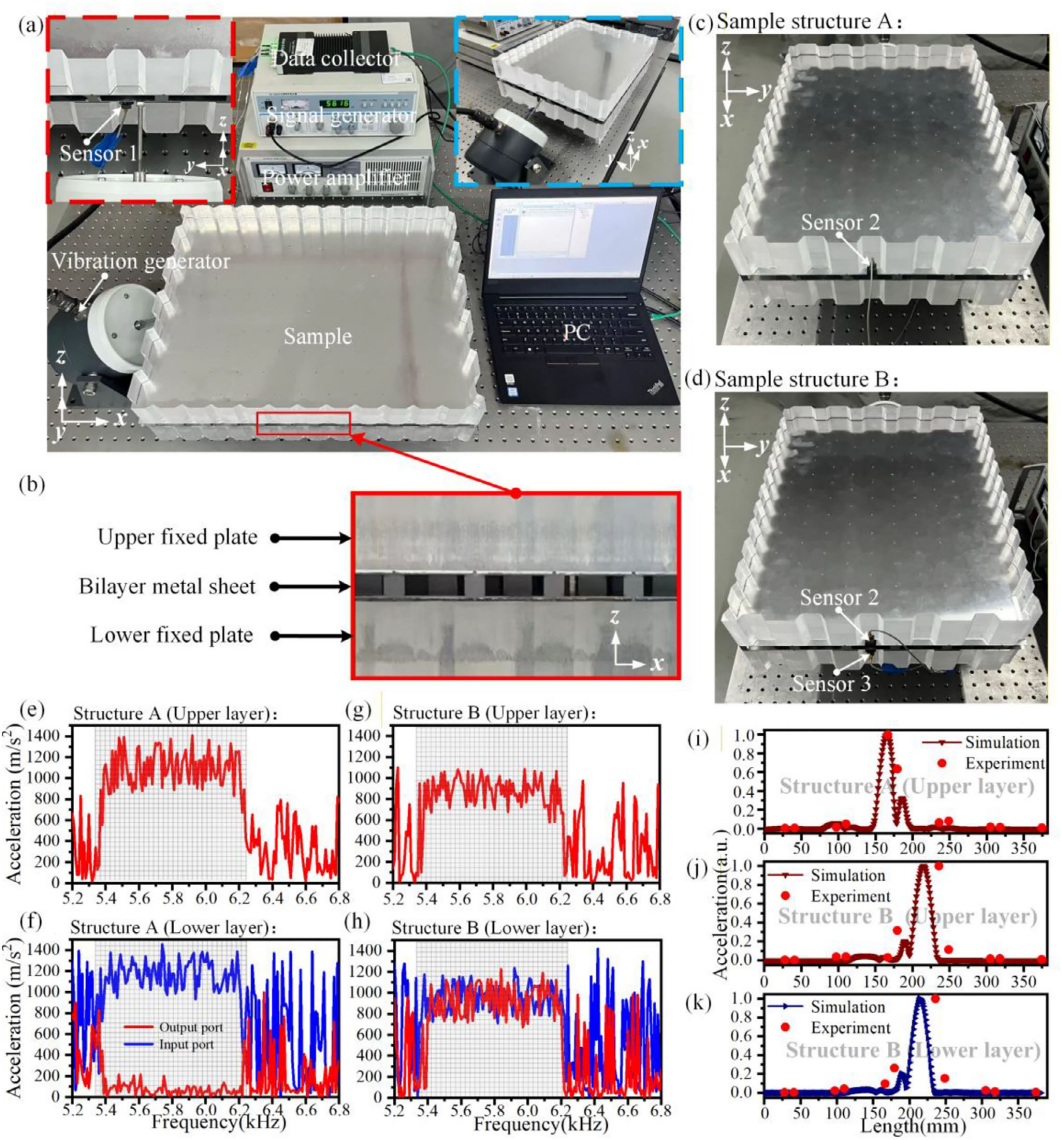
Experimental verification of interlayer conversion and beam splitting. (a) Experimental setup and arrangement. Top‐left inset: excitation end under vibrator excitation; top‐right inset: oblique view of the overall setup. (b) Composition and assembly of the sample. (c) Sample of structure A. (d) Sample of structure B. (e–h) The vibration acceleration in the upper and lower metal plates of structures A,B under different frequencies. (i–k) Transmission accuracy in the upper metal plate of structure A, and in the upper and lower metal plates of structure B.

Based on the simulated structures of structure A (Figure 4a) and structure B (Figure 6a), corresponding samples are fabricated, as shown in Figure 9c,d. Both samples consist of bilayer thin metal sheets clamped by fixed transparent acrylic plates. Figure 9b presents the detailed composition of the region highlighted by the enlarged red dashed box in Figure 9a. The structure is centrally symmetric, comprising an upper fixed plate, the bilayer metal sheets, and a lower fixed plate. Protrusions on metal sheets are in direct contact with the fixed plates, as illustrated in Figures 1a,c, and 9b. The fixed plates are fabricated from transparent organic acrylic resin, with a mass density of 1190 kg/m^3^, a Young's modulus of 3.3 GPa, and a Poisson's ratio of 0.40. Owing to their substantially larger volume and mass compared with the structure between them, the organic acrylic resin plates behave effectively as rigid bodies. Consequently, the contact interfaces between the protrusions and the acrylic plates are treated as fixed constraints, while the remaining parts of the metal sheets remain effectively free.

Subsequently, both samples are tested, with the overall experimental setup shown in Figure 9a. For structure A (Figure 9c and top‐left inset of Figure 9a), vibration acceleration sensors 1 and 2 (B&K Type 4508 B 001) are placed at the elastic wave incidence end and the output end of the thin metal plate, respectively. The vibration generator excited the edge of the lower thin metal plate. Sensor 1 measures the vibration acceleration at the incidence end, represented by the blue line in Figure 9f. Sensor 2 measures the vibration acceleration in the upper and lower layers at the output end, represented by the red lines in Figure 9e,f. In the upper layer, despite minor frequency shifts caused by machining, assembly, and measurement errors, the experimental trends closely match the frequency range of the topological interface modes. Measurements in these bands at the lower layer show negligible vibration acceleration, confirming efficient interlayer conversion of high‐performance elastic waves. The transmission accuracy at the upper output terminal was further characterized by scanning with sensor 2 (Figure 9i), revealing excellent agreement with simulation trends, despite minor deviations.

Similarly, measurements are performed on Structure B (Figure 9d). Sensor 1 is used to measure the vibration acceleration at the end of the incidence, while sensors 2 and 3 are placed at the output ends of the upper and lower layers, respectively. The measured vibration acceleration at the incidence end and at both output terminals under different excitation frequencies are shown in Figure 9g,h. The corresponding transmission accuracy at the upper and lower output terminals are presented in Figure 9j,k. A small deviation is observed between the peak positions obtained from the experimental measurements and those predicted by the simulations. This discrepancy can be attributed to several factors. First, boundary effects cause the peak positions at the output terminals to exhibit a certain degree of variation within a controllable range. Second, the limited size of the upper cover plate constrains sensor placement near the boundaries, restricting measurements to specific regions. In addition, fabrication tolerances and experimental measurement uncertainties can further contribute to deviations in the experimental results. Despite these factors, the experimental results show overall good agreement with the simulation predictions, and the observed trends are consistent with the expected behavior of the structure.

During the experiment, to ensure stable excitation, the exciter applies single‐frequency signals sequentially and records the response at each sensor. To enhance the stability and reliability of the experimental results, multiple measurements are conducted at each frequency, and the final results are obtained by averaging the measured data.

**FIGURE 10:**
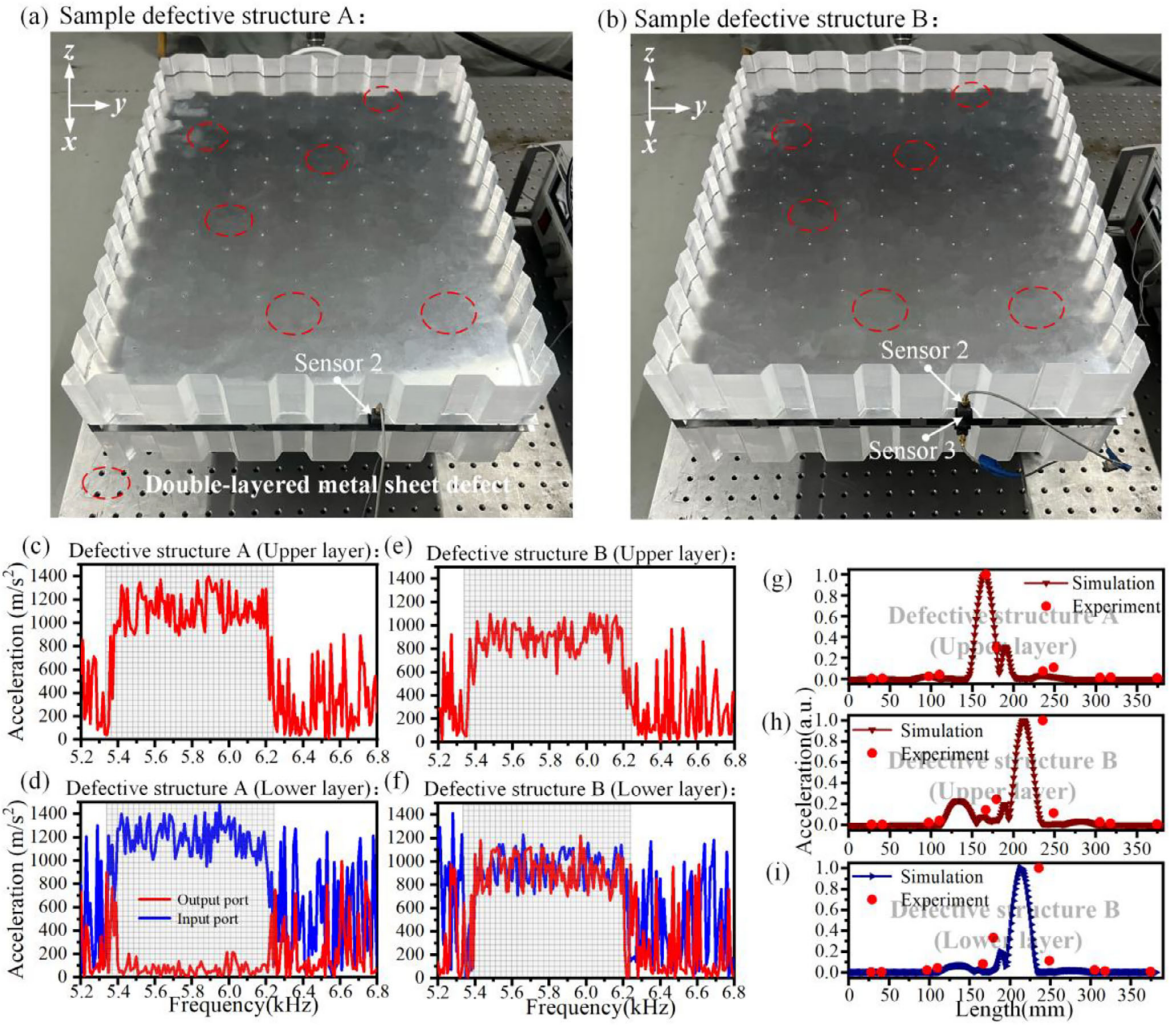
Experimental verification of high‐fault‐tolerance interlayer conversion and beam splitting. (a) Sample of defective structure A, with the red dashed circle indicating the missing coupled twisted triangular prism. (b) Sample of defective structure B. (c–f) The vibration acceleration in the upper and lower metal plates of defective structures A and B at different frequencies. (g–i) Transmission accuracy in the upper metal plate of defective structure A, the upper metal plate of defective structure B, and the lower metal plate of defective structure B.

Samples of defective structures A and B are also fabricated, as shown in Figure 10a,b, with the red dashed circles indicating missing portions of the couple‐twisting triangular prisms. Both samples are subsequently tested, and the experimental testing method is the same as above. For defective structure A, the vibration acceleration at incidence end and output end under the upper and lower layers are displayed in Figure 10c,d, and the transmission accuracy was also measured, as displayed in Figure 10g.

Similarly, defective structure B is also tested, the vibration acceleration at incidence end and output end under the upper and lower layers are displayed in Figure 10e,f, and the transmission accuracy are displayed in Figure 10h,i.

Furthermore, the experimental results measured at the output terminals of intact Structures A and B, as well as defective Structures A and B, are analyzed to investigate the influence of defects on the effective range Λ. Gaussian fitting is applied to the experimental data to obtain the corresponding fitting curves, from which the effective range Λ associated with 90% energy attenuation is extracted and summarized in Table 3. The results in Table 3 indicate that the presence of defects has a negligible effect on the transmission accuracy, thereby demonstrating the high fault‐tolerant capability of the proposed structures.

**TABLE 3:** Effective range (Λ) corresponding to 90% energy attenuation at the output terminals of intact and defective Structures A and B in the experiment.

Experimental Configuration	Output Terminal
Intact Structure A (Upper layer)	Λ = 1.075*a*
Defective structure A (Upper layer)	Λ = 1.162*a*
Intact Structure B (Upper layer)	Λ = 0.928*a*
Defective structure B (Upper layer)	Λ = 0.988*a*
Intact Structure B (Lower layer)	Λ = 1.071*a*
Defective structure B (Lower layer)	Λ = 1.014*a*

Although minor discrepancies exist between experimental and simulation results, the overall trends are in good agreement. The results demonstrate that the presence of structural defects does not affect interlayer conversion or beam splitting of high‐performance elastic waves, confirming the high‐fault‐tolerance of the BLEWTIs and further validating the study's findings.

## 4 Summary

In summary, this study demonstrates a series of BLEWTIs and their associated high‐performance interlayer converters and beam splitters, exhibiting remarkable fault tolerance. Band inversion and topological phase transitions are realized by rotating the coupled twisted triangular prisms between bilayer metal plates, giving rise to four distinct topological phases. This design strategy enables BLEWTIs with various domain walls, facilitating high‐performance interlayer conversion and beam splitting for elastic wave transmission with minimal loss and high precision. Experimental measurements on both intact and defective versions of structure A (interlayer conversion) and structure B (beam splitting) confirm that the transmission characteristics and cross‐layer accuracy are in excellent agreement with simulations, demonstrating that high‐performance elastic wave transmission can be maintained even in the presence of structural defects. This work establishes a new framework for designing BLEWTIs and offers significant potential for multilayer elastic wave devices, including multi‐channel transmitters, beam splitters, layer‐selective energy trapping, and information processing, while providing insights that are broadly applicable to other classical wave systems.

## Author Contributions

**Chengzhi Ma**: performed conceptualization, data curation, formal analysis, funding acquisition, investigation, methodology, software, validation, visualization, wrote the original draft, wrote the original draft and edited. **Zhiwei Song**: performed validation. **Zheyu Cheng**: performed wrote the original draft and edited. **Jiu Hui Wu**: performed resources. **Baile Zhang**: performed wrote the original draft and edited, funding acquisition, resources, supervision.

## Conflicts of Interest

The authors declare no conflict of interest.

## Data Availability

The data that support the findings of this study are available from the corresponding author upon reasonable request.
